# ECMO is associated with decreased hospital mortality in COVID-19 ARDS

**DOI:** 10.1038/s41598-024-64949-x

**Published:** 2024-06-27

**Authors:** Won-Young Kim, Sun-Young Jung, Jeong-Yeon Kim, Ganghee Chae, Junghyun Kim, Joon-Sung Joh, Tae Yun Park, Ae-Rin Baek, Yangjin Jegal, Chi Ryang Chung, Jinwoo Lee, Young-Jae Cho, Joo Hun Park, Jung Hwa Hwang, Jin Woo Song

**Affiliations:** 1 Division of Pulmonary and Critical Care Medicine, Department of Internal Medicine, Chung-Ang University Hospital, Chung-Ang University College of Medicine, Seoul, Republic of Korea; 2 College of Pharmacy, Chung-Ang University, Seoul, Republic of Korea; 3 Department of Pulmonary and Critical Care Medicine, Ulsan University Hospital, University of Ulsan College of Medicine, Ulsan, Republic of Korea; 4 Division of Pulmonary, Allergy and Critical Care Medicine, Department of Internal Medicine, Hallym University Dongtan Sacred Heart Hospital, Hallym University College of Medicine, Hwaseong, Republic of Korea; 5 Division of Pulmonary and Critical Care Medicine, Department of Internal Medicine, National Medical Center, Seoul, Republic of Korea; 6 Division of Pulmonary and Critical Care Medicine, Department of Internal Medicine, Seoul Metropolitan Government-Seoul National University Borame Medical Center, Seoul, Republic of Korea; 7 Division of Allergy and Pulmonology, Department of Internal Medicine, Soonchunhyang University Bucheon Hospital, Soonchunhyang University College of Medicine, Bucheon, Republic of Korea; 8 Department of Critical Care Medicine, Samsung Medical Center, Sungkyunkwan University School of Medicine, Seoul, Republic of Korea; 9 Division of Pulmonary and Critical Care Medicine, Department of Internal Medicine, Seoul National University College of Medicine, Seoul, Republic of Korea; 10 Division of Pulmonary and Critical Care Medicine, Department of Internal Medicine, Seoul National University Bundang Hospital, Seoul National University College of Medicine, Seongnam, Republic of Korea; 11 Department of Pulmonary and Critical Care Medicine, Ajou University Hospital, Ajou University School of Medicine, Suwon, Republic of Korea; 12 Department of Radiology, Soonchunhyang University Hospital, Soonchunhyang University College of Medicine, Seoul, Republic of Korea; 13 Department of Pulmonary and Critical Care Medicine, Asan Medical Center, University of Ulsan College of Medicine, Seoul, Republic of Korea

**Keywords:** Prognosis, Risk factors

## Abstract

This study determined whether compared to conventional mechanical ventilation (MV), extracorporeal membrane oxygenation (ECMO) is associated with decreased hospital mortality or fibrotic changes in patients with COVID-19 acute respiratory distress syndrome. A cohort of 72 patients treated with ECMO and 390 with conventional MV were analyzed (February 2020–December 2021). A target trial was emulated comparing the treatment strategies of initiating ECMO vs no ECMO within 7 days of MV in patients with a PaO_2_/FiO_2_ < 80 or a PaCO_2_ ≥ 60 mmHg. A total of 222 patients met the eligibility criteria for the emulated trial, among whom 42 initiated ECMO. ECMO was associated with a lower risk of hospital mortality (hazard ratio [HR], 0.56; 95% confidence interval [CI] 0.36–0.96). The risk was lower in patients who were younger (age < 70 years), had less comorbidities (Charlson comorbidity index < 2), underwent prone positioning before ECMO, and had driving pressures ≥ 15 cmH_2_O at inclusion. Furthermore, ECMO was associated with a lower risk of fibrotic changes (HR, 0.30; 95% CI 0.11–0.70). However, the finding was limited due to relatively small number of patients and differences in observability between the ECMO and conventional MV groups.

## Introduction

Acute respiratory distress syndrome (ARDS) is caused by direct or indirect lung injury resulting in multiorgan dysfunction, with an excess mortality of about 60% in the most severe forms^1^. Mechanical ventilation (MV) using lung-protective ventilation strategies is the mainstay of treatment^2^. In the most severe cases, however, ventilator-induced lung injury can still occur and adjuvant therapies may be required^3^. Extracorporeal membrane oxygenation (ECMO) can replace pulmonary gas exchange by providing oxygen and removing carbon dioxide from the blood, permitting MV to be performed at a lower intensity^4^. Accordingly, randomized trials and meta-analyses have supported the beneficial effects of ECMO for refractory ARDS^5–7^.

Retrospective cohort studies on patients with coronavirus disease 2019 (COVID-19) ARDS receiving ECMO reported 90-day mortality rates of 36.0–37.4%^8,9^. As the pandemic progressed, however, subsequent studies reported increasing mortality (51.9–60.1%) and longer durations of ECMO^10,11^. These studies were limited by patient selection and the lack of a proper control group. Recently, several studies conducted an emulated target trial using observational data and suggested that patients with COVID-19 ARDS may benefit more from ECMO than from MV^12–14^. However, these studies did not report data on the outcomes beyond mortality, such as lung damage and impaired lung function.

The present study aimed to compare the outcomes of patients with COVID-19 ARDS receiving ECMO vs conventional MV. The secondary objective was to determine whether ECMO was independently associated with decreased fibrotic changes.

## Methods

### Study setting and patient selection

This was a post-hoc analysis of a Korean multicenter registry consisting of two independent cohorts. The prospective cohort enrolled adult (age ≥ 18 years) patients with COVID-19 pneumonia (defined as positive real-time reverse transcription-polymerase chain reaction from a nasopharyngeal swab) who received MV and were admitted to one of the intensive care units (ICUs) at the eight tertiary or referral hospitals from July to December 2021. Patients were excluded if they had a previous diagnosis of interstitial lung disease. The retrospective cohort included patients who met the same criteria as were used in the prospective cohort at the 10 hospitals from February 2020 to September 2021. The local institutional review board of each hospital approved the study protocol (Institutional Review Board of Asan Medical Center on May 21, 2021; No. 2021-0769 and 2021-1353; Study title: A study on the clinical characteristics including severity of pulmonary fibrosis by COVID-19). The study was registered with the Clinical Research Informative Service (No. KCT0006312). Written informed consent was obtained from all participants or their next of kin in the prospective cohort but was waived in the retrospective cohort. The data were collected by intensivists or research nurses trained in critical care using an electronic case report form (iCReaT, https://icreat.nih.go.kr/icreat/). All consecutive patients prospectively or retrospectively registered in the dataset up to December 24, 2021, were analyzed. There were no overlapping patients between the two cohorts. Study procedures were followed in accordance with the ethical standards of the responsible committee on human experimentation and with the Helsinki Declaration of 1975.

### Outcomes and data collection

The primary outcome was hospital mortality. Fibrotic change was the secondary outcome. Demographic data included age, sex, body mass index (BMI), and comorbidities. Baseline characteristics at ICU admission included the type of oxygen support, sequential organ failure assessment (SOFA) score^15^, and laboratory findings. The dates of ICU admission, MV initiation, and ECMO initiation were retrieved. The treatment variables included corticosteroids, rescue therapies for ARDS (neuromuscular blocker, inhaled nitric oxide, and prone positioning), and renal replacement therapy. Arterial blood gas data and the daily mean values of respiratory rate, tidal volume, positive end-expiratory pressure (PEEP), and peak inspiratory pressure (PIP) were collected for the first 7 days of MV. Driving pressure and mechanical power were calculated as previously described^16,17^.

### Chest computed tomography (CT) scans and pulmonary function tests (PFTs)

In the prospective cohort, survivors were followed up in each hospital’s outpatient department one month after discharge where chest CT scans and PFTs were performed. For patients who died before discharge or were transferred to other hospitals, the last scans performed during the hospitalization were retrieved. The same criteria used in the prospective cohort were applied in the retrospective cohort. All CT images were assessed for the presence of fibrotic patterns (see Supplementary Appendix 1)^18^ by four thoracic radiologists (J.H.H., B.D.N., S.L., J.W.L.), one of them who had more than 20 years of experience (J.H.H.). To minimize inter-radiologist variability, a consensus meeting was held for sample cases before conducting further analyses; the differences in the assessment were resolved by consensus. A fibrotic change was defined as the presence of two or more fibrotic patterns. Spirometry was performed, and diffusing capacity (DLCO) by plethysmography was measured according to the European Respiratory Society/American Thoracic Society recommendations^19,20^.

### Statistical analysis

The data are reported as medians (interquartile ranges, IQRs) for continuous variables and as percentages for categorical variables. The outcome rates were compared by the incidence proportion and rate per 100 person-days with 95% confidence intervals (CIs). No power calculation was performed due to the post-hoc nature of the study.

A pragmatic randomized trial, which was similar to a per protocol analysis of the ECMO to rescue lung injury in severe ARDS (EOLIA) trial^6^, was emulated using the dataset. This robust approach has been described in previous studies^13,14^. Patients who received MV for ≤ 7 days with a PaO_2_/FiO_2_ < 80 or a PaCO_2_ ≥ 60 mmHg were eligible for the emulated trial. ECMO was utilized in all centers of current study; thus, all patients were considered as potential candidates for ECMO. An emulated trial was generated for each day from day 1 to day 7 of MV initiation. Among patients who met the eligibility criteria on day 1, those treated with ECMO were defined as the ECMO group, and those who did not initiate ECMO were defined as the conventional MV group. This method was repeated from day 2 to day 7 for patients who were alive, met the eligibility criteria, and did not initiate ECMO. In other words, a patient could meet the eligibility criteria and be included in the conventional MV group in several trials but only once in the ECMO group. The final cohort was constructed by combining the data from the 7 emulated trials.

Some patients in the conventional MV group eventually initiated ECMO and were artificially censored at ECMO initiation. To account for the dependent censoring resulting from this artificial censoring, inverse probability of censoring weighting (IPCW) analysis that adjusted for confounding measured at the start of each trial was conducted^21^. Baseline time-independent and -dependent covariates were used to estimate IPCW. Of these, PaO_2_/FiO_2_, tidal volume, PEEP, PIP, driving pressure, and mechanical power varied over time during follow-up. Missing values were estimated by multivariate imputation by chained equations, generating 10 datasets under the assumption of missing at random^22^.

The Cox proportional hazard regression was used to determine the associations between ECMO and hospital mortality or fibrotic change and estimated hazard ratios (HRs) with 95% CIs^23^. Given the duplicate patients in more than one trial and the use of IPCW, model-based variance estimators were not appropriate. The estimation of weights and the Cox model were repeated in non-parametric bootstrap with 200 resamples. CIs were estimated as the 2.5th and 97.5th percentiles of point estimates obtained from the bootstrap samples of the data. Four patients survived with ECMO and lung transplant, and this may have resulted in a decreased mortality or fibrotic changes in the ECMO group. Thus, the outcome analyses were conducted excluding these patients. In the IPCW-weighted final cohort, we performed weighted Cox regression analysis adjusting for imbalanced covariates between the groups (standardized mean difference of ≥ 0.20). Subgroup analyses investigated whether age, Charlson comorbidity index (CCI)^24^, prone positioning, severity of hypoxemia (PaO_2_/FiO_2_ at study inclusion) and driving pressure at study inclusion were potential effect modifiers. The cutoff values for age and CCI were based on the median values of the study patients. Based on a landmark study, a driving pressure ≥ 15 vs < 15 cmH_2_O was selected^25^. Three planned sensitivity analyses were performed. First, Cox analysis with fibrotic changes as the outcome was conducted including only patients who survived to hospital discharge. Second, a separate analysis investigated the association between ECMO and mortality including patients who survived with ECMO and lung transplant. Third, two distinct cohorts were analyzed by recruiting patients earlier or later in the pandemic. Thus, the potential effect of changes in the treatment period was assessed by further adjusting for study period during which there was a prominent SARS-CoV-2 strain circulating in Korea: wild type and Alpha (February 2020–June 2021) and Delta (July–December 2021).

Statistical significance was determined at a *P*-value of < 0.05 (two-tailed). All analyses were computed with SAS version 9.4 software (SAS Institute, Cary, NC, USA). The SAS codes are provided as Supplementary Appendix 2 for reference.

## Results

### Study population

**Figure 1:**
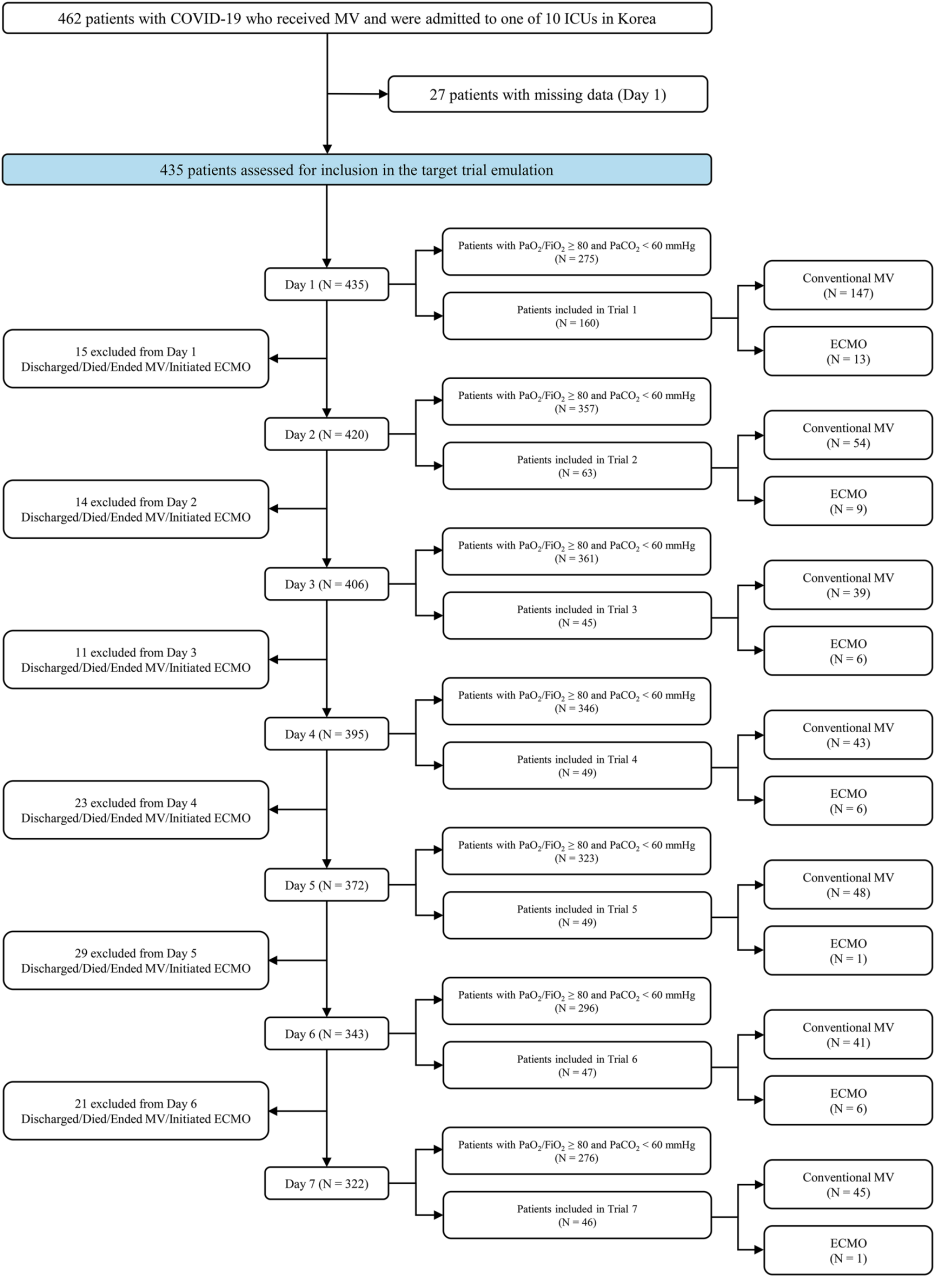
Study design and patient flow. The flow diagram shows the number of patients assessed for eligibility, the number of patients who met the criteria for the emulated trial, and the number of patients included in the ECMO and conventional MV groups during the first 7 days of MV. *COVID-19* coronavirus disease 2019, *ECMO* extracorporeal membrane oxygenation, *FiO*_2_ fraction of inspired oxygen, *ICU* intensive care unit, *MV* mechanical ventilation, *PaCO*_2_ arterial carbon dioxide tension, *PaO*_2_ arterial oxygen tension.

During the study period, 462 patients (72 ECMO and 390 conventional MV) were analyzed (Fig. 1). The median (IQR) duration of follow-up was 56 (29–92) days in the ECMO group and 28 (19–48) days in the conventional MV group. Since the primary outcome was observed during the hospitalization for all patients, there were no loss to follow-up. A total of 222 (48.1%) patients met the criteria for the emulated trial, among whom 42 (18.9%) initiated ECMO within 7 days of MV.

The baseline characteristics of patients included in the target trial emulation (original population) are shown in Table 1. The median (IQR) age was 71 (64–76) years, 140 (63.1%) were men, and the median (IQR) BMI was 25.1 (22.9–27.5) kg/m^2^ with a median (IQR) CCI of 1 (0–2) at ICU admission. Patients who were treated with ECMO were more likely to be younger, male, and have a higher BMI and a higher SOFA score. The median (IQR) PaO_2_/FiO_2_ in the ECMO and conventional MV groups was 78 (57–108) and 65 (56–74), respectively. From ICU admission, MV was initiated at a median (IQR) time of 0 (0–1) days in the ECMO group and 0 (0–2) days in the conventional MV group. The proportion of patients who received corticosteroids, neuromuscular blocker, or inhaled nitric oxide was similar between the groups. However, the ECMO group were more likely to receive renal replacement therapy, and the conventional MV group were more likely to receive prone positioning. ECMO was initiated at a median (IQR) time of 1 (0–3) days after MV initiation, and the median (IQR) duration was 17 (10–41) days. All prone positioning were performed before ECMO initiation. The ventilation parameters at study inclusion were similar between the groups, although respiratory rate, tidal volume, minute ventilation, PIP, driving pressure, and mechanical power values were significantly lower in the ECMO group in subsequent days (see Supplementary Table S1). After exclusion of the deceased patients, the median (IQR) duration of MV (25 [17–46] days vs 17 [10–44] days; *P* = 0.25), ICU length of stay (37 [28–48] days vs 27 [17–52] days; *P* = 0.45), and hospital length of stay (60 [38–85] days vs 44 [27–76] days; *P* = 0.20) did not differ between the groups. ECMO patients were more likely to undergo tracheostomy (59.5% vs 40.6%; *P* = 0.03).

**Table 1:** Baseline characteristics of patients included in the target trial emulation of ECMO vs conventional MV.

Characteristics	Original population				Final cohort			
	All	ECMO	Conventional MV	SMD	All	ECMO	Conventional MV	SMD
No. of patients	222	42	180		459	42	417	
Age, years	71 (64–76)	61 (49–68)	72 (66–77)	–1.00	72 (64–77)	61 (49–68)	73 (66–77)	–1.01
Sex				0.57				0.48
Male	140 (63.1)	35 (83.3)	105 (58.3)		296 (64.5)	35 (83.3)	261 (62.6)	
Female	82 (36.9)	7 (16.7)	75 (41.7)		163 (35.5)	7 (16.7)	156 (37.4)	
Body mass index, kg/m^2^	25.1 (22.9–27.5)	25.4 (22.8–27.4)	25.0 (22.9–27.6)	0.26	24.6 (22.8–26.7)	25.5 (22.8–27.4)	24.6 (22.8–26.6)	0.31
Missing data	2 (0.9)	1 (2.4)	1 (0.6)					
Charlson comorbidity index	1 (0–2)	1 (0–2)	1 (0–2)	–0.17	1 (0–2)	1 (0–2)	1 (0–2)	–0.09
Type of oxygen support				0.24				0.24
Mask with reservoir bag	139 (62.6)	28 (66.7)	111 (61.7)		310 (67.5)	28 (66.7)	282 (67.6)	
High-flow nasal cannula	38 (17.1)	8 (19.0)	30 (16.7)		67 (14.6)	8 (19.0)	59 (14.1)	
Intubated state	23 (10.4)	4 (9.5)	19 (10.6)		42 (9.2)	4 (9.5)	38 (9.1)	
SOFA score	4 (3–7)	4 (3–9)	4 (3–7)	0.23	4 (3–7)	4 (3–9)	4 (3–7)	0.31
Missing data	14 (6.3)	0	14 (7.8)					
Laboratory findings								
Lymphocyte, %	6.2 (4.0–10.5)	7.0 (4.1–10.5)	6.0 (3.9–10.3)	–0.03	5.7 (3.8–10.3)	7.0 (4.1–10.5)	5.7 (3.7–10.3)	–0.02
Missing data	3 (1.4)	0	3 (1.7)					
Platelet count, 1000/mm^3^	183 (139–257)	209 (146–288)	181 (139–247)	0.30	185 (139–260)	209 (146–288)	183 (139–252)	0.25
Missing data	4 (1.8)	0	4 (2.2)					
Total bilirubin, mg/dL	0.6 (0.4–0.8)	0.6 (0.5–0.8)	0.6 (0.4–0.9)	–0.03	0.6 (0.4–0.9)	0.6 (0.5–0.8)	0.6 (0.4–0.9)	0.001
Missing data	4 (1.8)	0	4 (2.2)					
Creatinine, mg/dL	0.8 (0.6–1.2)	0.8 (0.6–1.2)	0.8 (0.6–1.2)	0.23	0.8 (0.6–1.2)	0.8 (0.6–1.2)	0.8 (0.6–1.2)	0.11
Missing data	3 (1.4)	0	3 (1.7)					
PaO_2_/FiO_2_*	66 (57–77)	78 (57–108)	65 (56–74)	0.60	67 (57–77)	78 (57–108)	66 (57–76)	0.49
PaCO_2_, mmHg*	42 (35–51)	46 (37–55)	41 (34–48)	0.23	45 (38–56)	46 (37–55)	45 (38–57)	–0.06
Bicarbonate, mEq/L	22.6 (19.9–25.2)	22.5 (20.8–24.0)	22.8 (19.8–25.2)	–0.07	22.5 (19.8–25.2)	22.5 (20.8–24.0)	22.5 (19.8–25.2)	–0.02
Missing data	20 (9.0)	1 (2.4)	19 (10.6)					
Time from ICU admission to MV initiation, days	0 (0–2)	0 (0–1)	0 (0–2)	–0.24	1 (0–3)	0 (0–1)	1 (0–3)	–0.40
Corticosteroids	214 (96.4)	40 (95.2)	174 (96.7)	–0.07	449 (97.8)	40 (95.2)	409 (98.1)	–0.16
Neuromuscular blocker	188 (84.7)	35 (83.3)	153 (85.0)	0.05	417 (90.8)	36 (85.7)	381 (91.4)	–0.18
Missing data	4 (1.8)	2 (4.8)	2 (1.1)					
Inhaled nitric oxide	58 (26.1)	10 (23.8)	48 (26.7)	–0.07	173 (37.7)	10 (23.8)	163 (39.1)	–0.33
Prone positioning	121 (54.5)	18 (42.9)	103 (57.2)	–0.29	303 (66.0)	18 (42.9)	285 (68.3)	–0.53
Renal replacement therapy	61 (27.5)	19 (45.2)	42 (23.3)	0.47	122 (26.6)	19 (45.2)	103 (24.7)	0.44
MV parameters*								
Respiratory rate, breaths/min	24 (21–28)	26 (22–29)	24 (21–27)	0.27	26 (22–29)	26 (22–29)	26 (22–29)	–0.06
Tidal volume, mL/kg PBW	7.3 (6.3–8.4)	6.8 (5.9–7.6)	7.4 (6.4–8.5)	–0.48	7.4 (6.4–8.8)	6.8 (5.9–7.6)	7.4 (6.5–8.9)	–0.54
Minute ventilation, L/min^†^	10.4 (8.7–12.3)	11.2 (9.5–12.7)	10.3 (8.7–12.1)	0.21	11.4 (9.4–13.3)	11.2 (9.5–12.7)	11.4 (9.4–13.3)	–0.13
PEEP, cmH_2_O	10 (8–12)	10 (8–12)	10 (8–11)	0.07	10 (8–11)	10 (8–12)	10 (8–11)	0.25
PIP, cmH_2_O	26 (24–30)	26 (23–29)	27 (24–30)	–0.33	27 (24–31)	26 (23–29)	27 (24–31)	– 0.20
Missing data	5 (2.3)	1 (2.4)	4 (2.2)					
Driving pressure, cmH_2_O^‡^	16 (14–20)	15 (14–18)	16 (14–20)	–0.33	18 (14–22)	16 (14–18)	18 (14–22)	–0.26
Missing data	5 (2.3)	1 (2.4)	4 (2.2)					
Compliance, mL/cmH_2_O^§^	27.1 (21.0–32.5)	28.5 (22.5–33.8)	26.1 (20.1–32.5)	0.16	25.0 (19.4–32.5)	28.2 (22.5–33.8)	24.4 (18.9–32.4)	0.10
Missing data	5 (2.3)	1 (2.4)	4 (2.2)					
Mechanical power, J/min^¶^	32.2 (26.2–39.1)	30.8 (24.6–37.2)	32.2 (26.3–39.2)	–0.24	35.6 (27.9–43.5)	30.1 (24.6–37.2)	36.1 (28.3–43.6)	–0.41
Missing data	5 (2.3)	1 (2.4)	4 (2.2)					

Data are presented as medians (interquartile ranges) or percentages (including a category for missing data). Missing data are for the original population.

*ECMO* extracorporeal membrane oxygenation, *FiO*_2_ fraction of inspired oxygen, *MV* mechanical ventilation, *PaCO*_2_ arterial carbon dioxide tension, *PaO*_2_ arterial oxygen tension, *PBW* predicted body weight, *PEEP* positive end-expiratory pressure, *PIP* peak inspiratory pressure, *SMD* standardized mean difference, *SOFA* sequential organ failure assessment.

*Assessed on the day of study inclusion.

^†^Calculated as respiratory rate × tidal volume.

^‡^Calculated as PIP – PEEP.

^§^Calculated as tidal volume/driving pressure.

^¶^Calculated as 0.098 × tidal volume × respiratory rate × (PIP–1/2 × driving pressure).

### Chest CT scans and PFTs

From ICU admission, 14 (33.3%) ECMO and 65 (36.1%) conventional MV patients had a CT at a median (IQR) time of 62 (33–87) and 47 (25–64) days, respectively (see Supplementary Table S2). Traction bronchiectasis was the most frequent abnormality in both groups. Notably, 46.8% were classified as patients with fibrotic change. A PFT was done in 8 (19.0%) ECMO and 19 (10.6%) conventional MV patients (see Supplementary Table S2). Lung function, expressed by FEV_1_, FVC, and FEV_1_/FVC, was nearly normal, although 95.5% had a decreased DLCO (< 80% predicted).

### Outcomes

The emulated trial analysis constructed a final cohort with similar baseline characteristics compared to the original population (Table 1). The IPCW analysis also yielded a similar cohort, although there were residual imbalances despite weighting (see Supplementary Table S3). These variables were further adjusted in the regression analysis.

**Figure 2:**
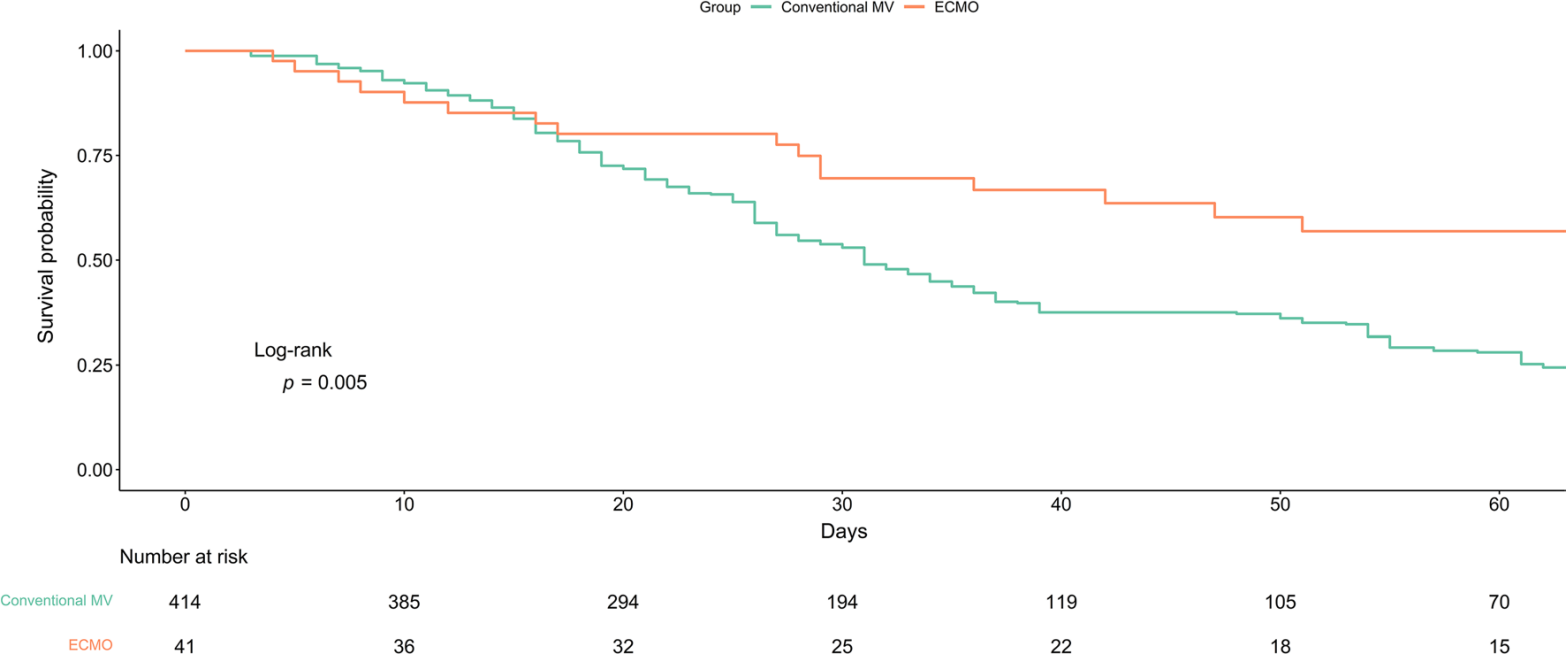
Survival from hospital admission to day 60 by study group. The median (interquartile range) time to death was 29 (12–73) days in the ECMO group and 26 (16–36) days in the conventional MV group. *ECMO* extracorporeal membrane oxygenation, *MV* mechanical ventilation.

Figure 2 shows the survival curves for the final cohort before weighting. The hospitalization mortality rate was 1.07 per 100 person-days (95% CI 0.74–1.42) among ECMO patients, compared with 1.92 per 100 person-days (95% CI 1.76–2.09) among conventional MV patients (Table 2). After weighting with adjustment for baseline imbalances, ECMO patients had a significantly lower risk of mortality (HR, 0.56; 95% CI 0.36–0.96). The survival curves for the final cohort after weighting are shown in Supplementary Fig. S1. The incidence rate of fibrotic change was 0.61 per 100 person-days (95% CI 0.33–0.94) in the ECMO group and 0.97 per 100 person-days (95% CI 0.82–1.12) in the conventional MV group (Table 2). After weighting with adjustment for baseline imbalances, the risk was significantly lower in the ECMO group (HR, 0.30; 95% CI 0.11–0.70).

**Table 2:** Incidence rates and hazard ratios comparing hospital mortality or fibrotic changes between ECMO and conventional MV.

Group	Incidence rate per 100 person-days (95% CI)	Hazard ratio (95% CI)
Hospital mortality		
Before weighting		
ECMO	1.07 (0.74–1.42)	0.54 (0.37–0.75)
Conventional MV	1.92 (1.76–2.09)	
IPCW-weighted		
ECMO	1.13 (0.75–1.58)	0.56 (0.36–0.96)*
Conventional MV	2.88 (2.51–3.43)	
Fibrotic changes		
Before weighting		
ECMO	0.61 (0.33–0.94)	0.44 (0.24–0.84)
Conventional MV	0.97 (0.82–1.12)	
IPCW-weighted		
ECMO	0.60 (0.31–0.94)	0.30 (0.11–0.70)*
Conventional MV	1.15 (0.96–1.34)	

*CI* confidence interval, *ECMO* extracorporeal membrane oxygenation, *IPCW* inverse probability of censoring weighting, *MV* mechanical ventilation.

*Adjusted for age, sex, body mass index, type of oxygen support, SOFA score, PaO_2_/FiO_2_, PaCO_2_, time from ICU admission to MV initiation, neuromuscular blocker, inhaled nitric oxide, prone positioning, renal replacement therapy, and mechanical power.

### Subgroup analyses

**Figure 3:**
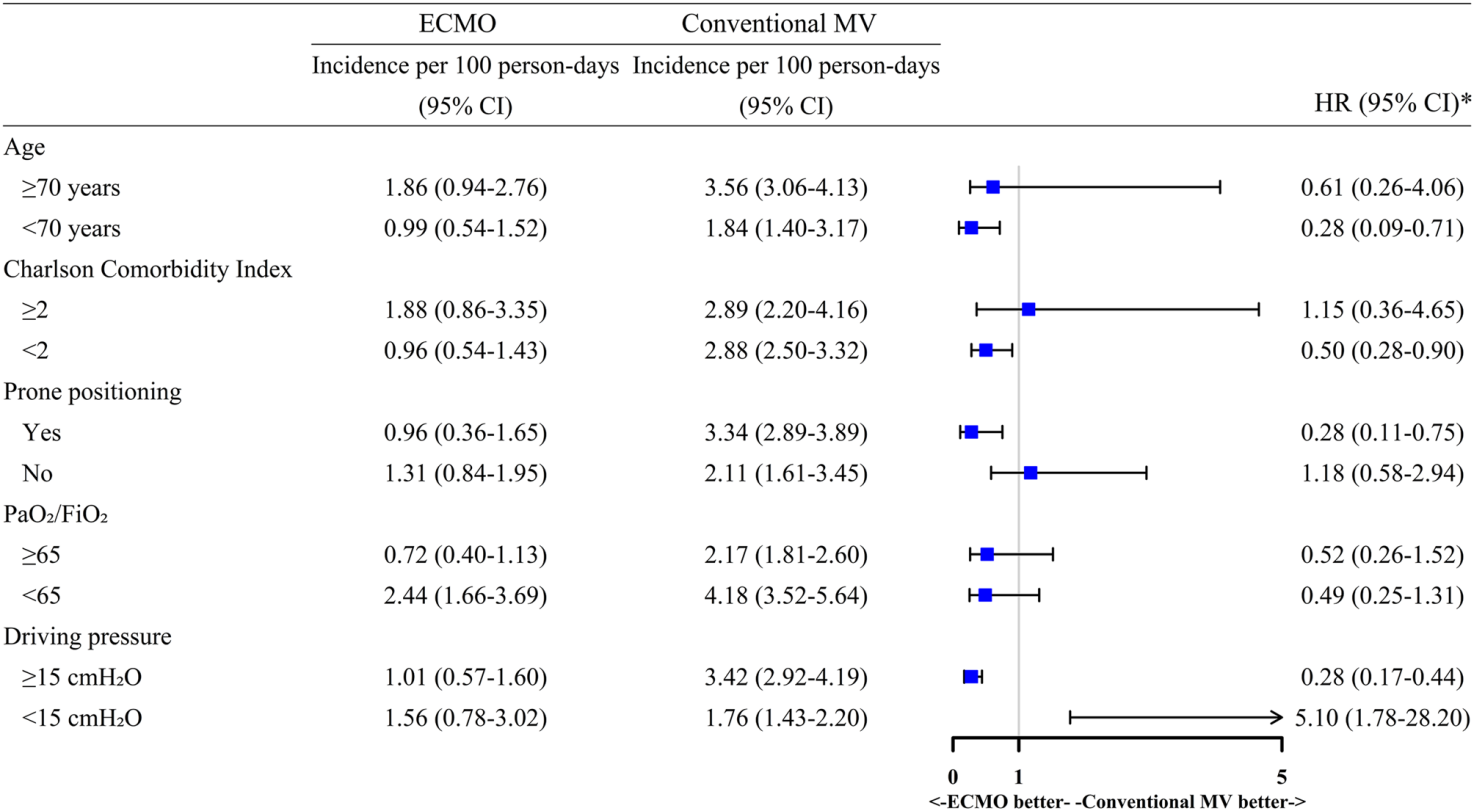
Hospital mortality between ECMO and conventional MV according to subgroup. The incidence rates and HRs (95% CIs) are estimated in the inverse probability of censoring weighted ECMO and conventional MV groups, stratified by age, Charlson Comorbidity Index, prone positioning, PaO_2_/FiO_2_ at study inclusion, and driving pressure at study inclusion. *Adjusted for age, sex, body mass index, type of oxygen support, SOFA score, PaO_2_/FiO_2_, PaCO_2_, time from ICU admission to MV initiation, neuromuscular blocker, inhaled nitric oxide, prone positioning, renal replacement therapy, and mechanical power. *CI* confidence interval, *ECMO* extracorporeal membrane oxygenation, *FiO*_2_ fraction of inspired oxygen, *HR* hazard ratio, *MV* mechanical ventilation, *PaO*_2_ arterial oxygen tension.

ECMO was associated with a significantly lower risk of hospital mortality in patients who were younger (age < 70 years; HR, 0.28; 95% CI 0.09–0.71), had less comorbidities (CCI < 2; HR, 0.50; 95% CI 0.28–0.90), underwent prone positioning (HR, 0.28; 95% CI 0.11–0.75), and had driving pressures ≥ 15 cmH_2_O (HR, 0.28; 95% CI 0.17–0.44; see Fig. 3 and Supplementary Table S4). Conversely, the risk of mortality was significantly higher in those who had driving pressures < 15 cmH_2_O (HR, 5.10; 95% CI 1.78–28.20). The mortality risk did not differ between the groups according to severity of hypoxemia.

### Sensitivity analyses

ECMO tended to be associated with decreased fibrotic change when the primary analysis was restricted to patients who survived to hospital discharge (HR, 0.29; 95% CI 0.05–1.14; see Supplementary Table S5). Similarly, the risk of hospital mortality was significantly lower in the ECMO group when analyses included patients who received ECMO and lung transplant (HR, 0.51; 95% CI 0.33–0.89; see Supplementary Table S6). When the primary analysis distinguished patients treated during the wild type and Alpha vs Delta period, ECMO was consistently associated with a significantly lower mortality risk (HR, 0.55; 95% CI 0.35–0.97) or fibrotic changes (HR, 0.18; 95% CI 0.03–0.55; see Supplementary Table S7).

## Discussion

This multicenter study revealed a lower risk of hospital mortality in patients with COVID-19 ARDS who were treated with ECMO compared to those treated with conventional MV. The findings were confined to younger patients with less comorbidities and exposure to higher driving pressures who underwent prone positioning. Moreover, ECMO was associated with a lower risk of fibrotic changes, albeit limited due to small number of patients and differences in observability between the groups.

The 52.4% hospital mortality rate of the ECMO group (conventional MV group: 60.0%) corroborates the 58.6% mortality rate reported in recent studies from the Asia–Pacific regions^26^. A relatively high mortality compared to those in previous studies of COVID-19 and non-COVID-19 ARDS^6,8,9^ may be explained by older patients treated with ECMO in the current study. Moreover, it is possible that over 95% of the patients who received corticosteroids but eventually progressed to severe ARDS requiring ECMO may represent steroid-resistant phenotypes^27^. In Korea, ECMO use is neither regulated nor restricted, resulting in some hospitals with small annual case volumes. However, data from the international registry suggested that ECMO was beneficial only when performed in high volume centers with well-organized ECMO services^10,28^. The time on non-invasive ventilation prior to intubation is also considered a prognostic factor^29^. However, we could not assess whether prolonged non-invasive ventilation and intubation delay is associated with outcomes because the median time from ICU admission to MV initiation was less than a day. Finally, the median ECMO duration of 17 days was comparable to those in previous studies^10,11^. The longer duration of ECMO in COVID-19 ARDS compared to non-COVID-19 ARDS may be due to higher rates of ECMO-associated complications, such as major bleeding, thromboembolic events, and ventilator-associated pneumonia^9–11,29^, although its impact on survival is unclear. Nonetheless, the study demonstrated that ECMO was associated with decreased mortality when used in patients with a PaO_2_/FiO_2_ < 80 or a PaCO_2_ ≥ 60 mmHg within the first 7 days of MV.

ECMO was found to be associated with a lower risk of hospital mortality in selected patients with younger age, less comorbidities, and exposure to higher driving pressures. These results corroborate a recent emulated target trial, although ECMO was also associated with decreased mortality in COVID-19 patients with age > 65 years, hypertension, obesity, diabetes, and a PaO_2_/FiO_2_ ≥ 80 and < 120, suggesting a potential role of ECMO for older patients with comorbidities and less severe hypoxemia^13^. Another emulated target trial found that ECMO was associated with a lower risk of mortality in COVID-19 patients with more severe hypoxemia (PaO_2_/FiO_2_ ≤ 65)^14^. In the current study, however, the mortality risk did not differ between the groups with a PaO_2_/FiO_2_ ≥ 65 vs < 65. These findings question whether ECMO should be limited to patients with COVID-19 ARDS who comply with recommendations from the extracorporeal life support organization guidelines^30^, which are largely based on the results of a randomized trial in those with non-COVID-19 ARDS^6^. Further studies are required regarding patient selection for ECMO in COVID-19 ARDS.

Prone positioning before ECMO was associated with a lower mortality risk. Several studies have shown that, despite refractory hypoxemia, proned patients had lower plateau and driving pressures before ECMO implementation, indicating that prone positioning before ECMO may protect the lungs from ventilator-induced lung injuries^31,32^. A recent meta-analysis also showed an improved survival when prone positioning was used during ECMO in patients with ARDS, including COVID-19^33^. However, no patient underwent prone positioning on ECMO in the present study. Numerous data showed that ECMO mortality increased after the first wave^10,11^. Nonetheless, ECMO was associated with decreased mortality compared to conventional MV even after adjustment for the early and late stages of the pandemic.

Patients with COVID-19 are more likely to be hospitalized and have longer hospitalizations with a higher likelihood of developing ARDS than those with other acute respiratory diseases^34^. These findings may be explained by the distinct pulmonary pathology of severe COVID-19, such as severe endothelial injury, disrupted cell membranes, and widespread thrombosis with microangiopathy^35^. Previous studies that performed chest CT scans during follow-up of COVID-19 survivors found that fibrosis was present in 23.6–49.1% of patients at three months after discharge^36,37^. These corroborate the current study (46.8%), even higher than those of survivors with other viral pneumonias^38,39^. Despite a nearly normalized FEV_1_ and FVC, most patients had an impaired DLCO at follow-up, indicating residual lung damage. COVID-19 ARDS is characterized by prolonged MV and ECMO durations and ICU and hospital lengths of stay^40^. MV days and ICU and hospital lengths of stay were similar between the ECMO and conventional MV groups. However, the risk of fibrotic change was lower in patients who were treated with ECMO. It is unclear whether the post-acute lung sequelae of COVID-19 derive from disease-specific mechanisms or ventilator-induced lung injury. The latter is supported by the finding that more protective ventilation was applied during ECMO to the study patients, which could have partly prevented progressive lung injury.

The present study has several limitations. First, the observational data cannot confirm a causal relationship between ECMO use and mortality or fibrotic changes. In addition, there were differences in baseline characteristics between the ECMO and conventional MV groups. Despite methodological techniques to emulate a randomized trial and adjust for imbalanced covariates, the possibility of unmeasured confounders remains. Second, the number of patients was relatively small. Moreover, the assessment of fibrotic changes, including subgroup analyses, was limited due to lack of CT scans in a considerable proportion of patients. Third, missing data and imputation of missing values might have biased the results. Fourth, there were no specific recommendations on the initiation and management of ECMO, and these could have differed between the centers. Fifth, it was not feasible to evaluate to what extent residual lung impairment is COVID-19-related because baseline values (presence of chronic lung disease, CT scans, or PFTs) were not available. Furthermore, the reversibility of fibrotic changes is unknown due to the short-term follow-up. Sixth, this study included many older patients (age > 70 years, 45.0% of the initial cohort), reflecting the high rate of ICU admission among older patients in Korea. Seventh, unequal follow-up between the groups may introduce bias into analyses due to differences in observability of study outcomes. However, when calculating the absolute percentage without considering follow-up duration, both fibrotic change and hospital mortality rates were lower in the ECMO group (see Supplementary Table S8).

In conclusion, ECMO was independently associated with decreased hospital mortality in patients with COVID-19 ARDS. Age and comorbidities, as well as exposure to higher driving pressures or when prone positioning was performed before ECMO should be considered when deciding to implement ECMO in these patients. Additional studies refining the indications of ECMO for COVID-19 patients are warranted. The finding that ECMO was associated with a lower risk of fibrotic changes should be interpreted with caution due to the aforementioned limitations. Protective ventilation strategies using ECMO may prevent ventilator-associated lung sequelae. Data on long-term follow-up of these patients are needed to confirm whether these sequelae persist.

### Supplementary Information

Supplementary Information.

## Data Availability

All data generated or analyzed during this study are included in this published article (and its Supplementary Information files).
