# Supplementary material for: ECMO is associated with decreased hospital mortality in COVID-19 ARDS
Source: Sci Rep. 2024 Jun 27;14:14835. doi: 10.1038/s41598-024-64949-x (PMC11211457; doi:10.1038/s41598-024-64949-x)
Supplement: Supplementary file 1 — Supplementary Information. [file 41598_2024_64949_MOESM1_ESM.pdf]

Supplementary Information

ECMO is associated with decreased hospital mortality in COVID-19 ARDS

Scientific Reports

Won-Young Kim[1], Sun-Young Jung[2], Jeong-Yeon Kim[2], Ganghee Chae[3], Junghyun Kim[4], Joon-Sung Joh[5], Tae Yun Park[6], Ae-Rin Baek[7], Yangjin Jegal[3], Chi Ryang Chung[8], Jinwoo Lee[9], Young-Jae Cho[10], Joo Hun Park[11], Jung Hwa Hwang[12], and Jin Woo Song[13]

[1]Division of Pulmonary and Critical Care Medicine, Department of Internal Medicine, Chung-Ang University Hospital, Chung-Ang University College of Medicine, Seoul, Republic of Korea. [2]College of Pharmacy, Chung-Ang University, Seoul, Republic of Korea. [3]Department of Pulmonary and Critical Care Medicine, Ulsan University Hospital, University of Ulsan College of Medicine, Ulsan, Republic of Korea. [4]Division of Pulmonary, Allergy and Critical Care Medicine, Department of Internal Medicine, Hallym University Dongtan Sacred Heart Hospital, Hallym University College of Medicine, Hwaseong, Republic of Korea. [5]Division of Pulmonary and Critical Care Medicine, Department of Internal Medicine, National Medical Center, Seoul, Republic of Korea. [6]Division of Pulmonary and Critical Care Medicine, Department of Internal Medicine, Seoul Metropolitan Government-Seoul National University Borame Medical Center, Seoul, Republic of Korea. [7]Division of Allergy and Pulmonology, Department of Internal Medicine, Soonchunhyang University Bucheon Hospital, Soonchunhyang University College of Medicine, Bucheon, Republic of Korea. [8]Department of Critical Care Medicine, Samsung Medical Center, Sungkyunkwan University School of Medicine, Seoul, Republic of Korea. [9]Division of Pulmonary and Critical Care Medicine, Department of Internal Medicine, Seoul National University College of Medicine, Seoul, Republic of Korea. [10]Division of Pulmonary and Critical Care Medicine, Department of Internal Medicine, Seoul National University Bundang Hospital, Seoul National University College of Medicine, Seongnam, Republic of Korea. [11]Department of Pulmonary and Critical Care Medicine, Ajou University Hospital, Ajou University School of Medicine, Suwon, Republic of Korea. [12]Department of Radiology, Soonchunhyang University Hospital, Soonchunhyang University College of Medicine, Seoul, Republic of Korea. [13]Department of Pulmonary and Critical Care Medicine, Asan Medical Center, University of Ulsan College of Medicine, Seoul, Republic of Korea.

Supplementary Appendix 1. Assessment of fibrotic patterns on chest CT.

Supplementary Appendix 2. SAS codes used for multiple imputation, bootstrap, IPCW modeling, and Cox regression analysis.

Supplementary Table S1. Ventilation parameters in the ECMO and conventional MV groups during the first three days of study inclusion.

Supplementary Table S2. Chest CT scans and pulmonary function tests in the ECMO and

conventional MV groups at follow-up.

Supplementary Table S3. Baseline characteristics of IPCW-weighted cohort.

Supplementary Table S4. Incidence rates and hazard ratios comparing hospital mortality between ECMO and conventional MV according to subgroup.

Supplementary Table S5. Incidence rates and hazard ratios comparing fibrotic changes between ECMO and conventional MV, in the model including only patients who survived to hospital discharge.

Supplementary Table S6. Incidence rates and hazard ratios comparing hospital mortality between ECMO and conventional MV, in the model including patients who received ECMO and lung transplant.

Supplementary Table S7. Incidence rates and hazard ratios comparing hospital mortality or fibrotic changes between ECMO and conventional MV, in the model further adjusted for the wild type and Alpha/Delta periods.

Supplementary Table S8. Hospital mortality and fibrotic changes between the ECMO and conventional MV groups.

Supplementary Figure S1. Survival from hospital admission to day 60 by study group in the IPCW-weighted cohort.

Supplementary Appendix 1. Assessment of fibrotic patterns on chest CT.

Variable	Definition
Reticulation	A network of fine lines; often irregularly spaced, with a mixture of thick and thin lines
Architectural distortion	A condition in which the anatomical structures of the lungs, such as bronchi, blood vessels, interlobular fissures, and interlobular septum, are abnormally distorted due to lung disease
Traction bronchiectasis	Irregular bronchial and bronchiolar dilatation caused by retractile fibrosis in the surrounding lung parenchyma; predominantly seen in the periphery of the lungs, and affected airways typically have an irregular varicose appearance; conglomerated lesion can resemble honeycombing, particularly when it predominates at the lung bases
Honeycombing	Typically located in the dorsal, basal, and subpleural regions of the lung; clustered, thick-walled cystic spaces of similar diameters, generally measuring between 3–5 mm, but occasionally up to 25 mm in size

CT: computed tomography.

Supplementary Appendix 2. SAS codes used for multiple imputation, bootstrap, IPCW modeling, and Cox regression analysis.

```
/*************************************************************************
* Multiple imputation
*************************************************************************/

/* Imputation Methods:
    DISCRIM Specifies the discriminant function method
    LOGISTIC Specifies the logistic regression method
    REG Specifies the regression method
    REGPMM Specifies the predictive mean matching method */

/* Imputation done using PROC MI */
proc mi data= Ecmo_mv nimpute=10 out= Ecmo_mv_mi seed=1234 ;
      /* Specify categorical variables for imputation */
      class Oxygen_type MV_mode NMB ;
      /* Specify imputation methods for each variable */
      fcs logistic(Oxygen_type MV_mode NMB / likelihood=augment link=glogit DETAILS)
            reg(/* baseline covariates */
            Age BMI SAPS2 SOFA Lymph_percent PLT Tbil Cr pH HCO3
            /* time-dependent covariates */
            MV_D1_PaCO2 MV_D1_PaO2 MV_D1_FiO2 MV_D1_RR MV_D1_TV MV_D1_PIP MV_D1_PEEP
            MV_D2_PaCO2 MV_D2_PaO2 MV_D2_FiO2 MV_D2_RR MV_D2_TV MV_D2_PIP MV_D2_PEEP
            MV_D3_PaCO2 MV_D3_PaO2 MV_D3_FiO2 MV_D3_RR MV_D3_TV MV_D3_PIP MV_D3_PEEP
            MV_D4_PaCO2 MV_D4_PaO2 MV_D4_FiO2 MV_D4_RR MV_D4_TV MV_D4_PIP MV_D4_PEEP
            MV_D5_PaCO2 MV_D5_PaO2 MV_D5_FiO2 MV_D5_RR MV_D5_TV MV_D5_PIP MV_D5_PEEP
            MV_D6_PaCO2 MV_D6_PaO2 MV_D6_FiO2 MV_D6_RR MV_D6_TV MV_D6_PIP MV_D6_PEEP
            MV_D7_PaCO2 MV_D7_PaO2 MV_D7_FiO2 MV_D7_RR MV_D7_TV MV_D7_PIP MV_D7_PEEP
            MV_D1_TV_PBW MV_D1_MV MV_D1_driving_pr MV_D1_compliance MV_D1_MP
            MV_D2_TV_PBW MV_D2_MV MV_D2_driving_pr MV_D2_compliance MV_D2_MP
            MV_D3_TV_PBW MV_D3_MV MV_D3_driving_pr MV_D3_compliance MV_D3_MP
            MV_D4_TV_PBW MV_D4_MV MV_D4_driving_pr MV_D4_compliance MV_D4_MP
            MV_D5_TV_PBW MV_D5_MV MV_D5_driving_pr MV_D5_compliance MV_D5_MP
            MV_D6_TV_PBW MV_D6_MV MV_D6_driving_pr MV_D6_compliance MV_D6_MP
            MV_D7_TV_PBW MV_D7_MV MV_D7_driving_pr MV_D7_compliance MV_D7_MP
             / DETAILS);
      /* Specify variables for imputation */
      var Oxygen_type MV_mode NMB Age BMI SAPS2 SOFA Lymph_percent PLT Tbil Cr pH HCO3
            MV_D1_PaCO2 MV_D1_PaO2 MV_D1_FiO2 MV_D1_RR MV_D1_TV MV_D1_PIP MV_D1_PEEP
            MV_D2_PaCO2 MV_D2_PaO2 MV_D2_FiO2 MV_D2_RR MV_D2_TV MV_D2_PIP MV_D2_PEEP
            MV_D3_PaCO2 MV_D3_PaO2 MV_D3_FiO2 MV_D3_RR MV_D3_TV MV_D3_PIP MV_D3_PEEP
            MV_D4_PaCO2 MV_D4_PaO2 MV_D4_FiO2 MV_D4_RR MV_D4_TV MV_D4_PIP MV_D4_PEEP
            MV_D5_PaCO2 MV_D5_PaO2 MV_D5_FiO2 MV_D5_RR MV_D5_TV MV_D5_PIP MV_D5_PEEP
            MV_D6_PaCO2 MV_D6_PaO2 MV_D6_FiO2 MV_D6_RR MV_D6_TV MV_D6_PIP MV_D6_PEEP
            MV_D7_PaCO2 MV_D7_PaO2 MV_D7_FiO2 MV_D7_RR MV_D7_TV MV_D7_PIP MV_D7_PEEP
            MV_D1_TV_PBW MV_D1_MV MV_D1_driving_pr MV_D1_compliance MV_D1_MP
            MV_D2_TV_PBW MV_D2_MV MV_D2_driving_pr MV_D2_compliance MV_D2_MP
            MV_D3_TV_PBW MV_D3_MV MV_D3_driving_pr MV_D3_compliance MV_D3_MP
            MV_D4_TV_PBW MV_D4_MV MV_D4_driving_pr MV_D4_compliance MV_D4_MP
            MV_D5_TV_PBW MV_D5_MV MV_D5_driving_pr MV_D5_compliance MV_D5_MP
            MV_D6_TV_PBW MV_D6_MV MV_D6_driving_pr MV_D6_compliance MV_D6_MP
            MV_D7_TV_PBW MV_D7_MV MV_D7_driving_pr MV_D7_compliance MV_D7_MP;
run;
```

```
/* Tabulation of Imputed Results */
proc tabulate data= Ecmo_mv_mi;
      /* Specify variables for tabulation */
      var Oxygen_type MV_mode NMB Age BMI SAPS2 SOFA Lymph_percent PLT Tbil Cr pH HCO3
             MV_D1_PaCO2 MV_D1_PaO2 MV_D1_FiO2 MV_D1_RR MV_D1_TV MV_D1_PIP MV_D1_PEEP
             MV_D2_PaCO2 MV_D2_PaO2 MV_D2_FiO2 MV_D2_RR MV_D2_TV MV_D2_PIP MV_D2_PEEP
             MV_D3_PaCO2 MV_D3_PaO2 MV_D3_FiO2 MV_D3_RR MV_D3_TV MV_D3_PIP MV_D3_PEEP
             MV_D4_PaCO2 MV_D4_PaO2 MV_D4_FiO2 MV_D4_RR MV_D4_TV MV_D4_PIP MV_D4_PEEP
             MV_D5_PaCO2 MV_D5_PaO2 MV_D5_FiO2 MV_D5_RR MV_D5_TV MV_D5_PIP MV_D5_PEEP
             MV_D6_PaCO2 MV_D6_PaO2 MV_D6_FiO2 MV_D6_RR MV_D6_TV MV_D6_PIP MV_D6_PEEP
             MV_D7_PaCO2 MV_D7_PaO2 MV_D7_FiO2 MV_D7_RR MV_D7_TV MV_D7_PIP MV_D7_PEEP
             MV_D1_TV_PBW MV_D1_MV MV_D1_driving_pr MV_D1_compliance MV_D1_MP
             MV_D2_TV_PBW MV_D2_MV MV_D2_driving_pr MV_D2_compliance MV_D2_MP
             MV_D3_TV_PBW MV_D3_MV MV_D3_driving_pr MV_D3_compliance MV_D3_MP
             MV_D4_TV_PBW MV_D4_MV MV_D4_driving_pr MV_D4_compliance MV_D4_MP
             MV_D5_TV_PBW MV_D5_MV MV_D5_driving_pr MV_D5_compliance MV_D5_MP
             MV_D6_TV_PBW MV_D6_MV MV_D6_driving_pr MV_D6_compliance MV_D6_MP
             MV_D7_TV_PBW MV_D7_MV MV_D7_driving_pr MV_D7_compliance MV_D7_MP;
      /* Specify classification variables */
      class No Name ;
      /* Define table structure */
      tables No*Name,
             ( Oxygen_type MV_mode NMB Age BMI SAPS2 SOFA Lymph_percent PLT Tbil Cr pH HCO3
             MV_D1_PaCO2 MV_D1_PaO2 MV_D1_FiO2 MV_D1_RR MV_D1_TV MV_D1_PIP MV_D1_PEEP
             MV_D2_PaCO2 MV_D2_PaO2 MV_D2_FiO2 MV_D2_RR MV_D2_TV MV_D2_PIP MV_D2_PEEP
             MV_D3_PaCO2 MV_D3_PaO2 MV_D3_FiO2 MV_D3_RR MV_D3_TV MV_D3_PIP MV_D3_PEEP
             MV_D4_PaCO2 MV_D4_PaO2 MV_D4_FiO2 MV_D4_RR MV_D4_TV MV_D4_PIP MV_D4_PEEP
             MV_D5_PaCO2 MV_D5_PaO2 MV_D5_FiO2 MV_D5_RR MV_D5_TV MV_D5_PIP MV_D5_PEEP
             MV_D6_PaCO2 MV_D6_PaO2 MV_D6_FiO2 MV_D6_RR MV_D6_TV MV_D6_PIP MV_D6_PEEP
             MV_D7_PaCO2 MV_D7_PaO2 MV_D7_FiO2 MV_D7_RR MV_D7_TV MV_D7_PIP MV_D7_PEEP
             MV_D1_TV_PBW MV_D1_MV MV_D1_driving_pr MV_D1_compliance MV_D1_MP
             MV_D2_TV_PBW MV_D2_MV MV_D2_driving_pr MV_D2_compliance MV_D2_MP
             MV_D3_TV_PBW MV_D3_MV MV_D3_driving_pr MV_D3_compliance MV_D3_MP
             MV_D4_TV_PBW MV_D4_MV MV_D4_driving_pr MV_D4_compliance MV_D4_MP
             MV_D5_TV_PBW MV_D5_MV MV_D5_driving_pr MV_D5_compliance MV_D5_MP
             MV_D6_TV_PBW MV_D6_MV MV_D6_driving_pr MV_D6_compliance MV_D6_MP
             MV_D7_TV_PBW MV_D7_MV MV_D7_driving_pr MV_D7_compliance MV_D7_MP)
             *median;
run;

/* Define Exclusion and Control Criteria based on Multiple Imputation Median Results */


/***************************************************************************
* Bootstrap
* Generating 200 bootstrap replications
***************************************************************************/

%let rep=200; /* Number of bootstrap replications */

proc surveyselect data= ipcw out=boot_sample
   seed=12345
   method=urs samprate=1 outhits /*Unrestricted random sampling*/
   rep=&rep.;
run;
```

```
/***********************************************************************
* IPCW (Inverse Probability of Censoring Weighting)
************************************************************************/

/* Censoring Weighting: */

/* Perform logistic regression for censoring */
proc logistic data=boot_sample;
        by Replicate; /* Perform the analysis for each bootstrap */
        class Hosp_mortality NO Sex DM HTN Cerebrovascular Liver Kidney Malignancy Lung
        Oxygen_type Steroid NMB Inhaled_NO Prone RRT ;
        model censor (ref='1') = trt Hosp_mortality Age Sex DM HTN Cerebrovascular Liver
        Kidney Malignancy Lung Oxygen_type pH HCO3 Steroid NMB Inhaled_NO Prone RRT
        MV_mode BMI CCI SOFA Lymph_percent PLT Tbil Cr ICU_adm_MV_start / firth rl ;
        OUTPUT OUT=PREDCEN0 PRED=PREDCEN0;
run;

/* Perform logistic regression for censoring with time-dependent covariates */
proc logistic data=boot_sample;
        by Replicate; /* Perform the analysis for each bootstrap */
        class Hosp_mortality NO Sex DM HTN   Cerebrovascular Liver   Kidney Malignancy Lung
        Oxygen_type Steroid NMB Inhaled_NO Prone RRT ;
        model censor (ref='1') = trt Hosp_mortality Age Sex DM HTN Cerebrovascular Liver
       Kidney Malignancy Lung Oxygen_type   pH HCO3 Steroid NMB Inhaled_NO Prone RRT
       MV_mode BMI CCI SOFA Lymph_percent PLT Tbil Cr ICU_adm_MV_start
        /* + time-dependent covariates*/
        MV_PaCO2 MV_PaO2 MV_FiO2 MV_RR   MV_PIP MV_TV_PBW MV_MV
        MV_driving_pr MV_compliance MV_MP / firth rl ;
        OUTPUT OUT=PREDCEN1 PRED=PREDCEN1;
run;

/* Calculate ratio of probabilities for censoring */
proc sql;
        create table PREDCEN as
        select distinct *, PREDCEN0/ PREDCEN1 as PREDCEN
        from PREDCEN1 (keep=replicate no day PREDCEN1)
        natural full join
        PREDCEN0 (keep=replicate no day PREDCEN0)
        order replicate, no, day     ;
quit;

/* Merge original dataset with calculated probabilities */
proc sql;
        create table ipcw_bootstrap as
        select distinct *
        from boot_sample as a
        left join PREDCEN as b
        on a.Replicate = b.Replicate and a.no = b.no and a.day = b.day ;
quit;

/***********************************************************************
* Cox proportional hazards regression model
************************************************************************/
/* Perform Proportional Hazards Regression (PHREG) */

proc phreg data = ipcw_bootstrap ;
```

```
            by replicate; /* Perform the analysis for each bootstrap */
            weight PREDCEN; /* Apply inverse probability of censoring weighting */
            class trt(ref="0");
            model FU * Hosp_mortality(0) = trt Age Sex BMI Oxygen_type SOFA ICU_adm_MV_start
            Inhaled_NO Prone RRT MV_PF MV_PaCO2 NMB MV_MP / eventcode = 1 rl;
            ods output ParameterEstimates=cox_out ;
run;

/* Macro to calculate 95% confidence intervals (CI) for hazard ratios (HR)*/
%macro cox(var=); /*list of explanatory variables of interest*/

            proc univariate data=cox_out noprint;
                        var estimate;
                        where parameter="&var";
                        output out=&var. pctlpts=2.5 97.5 pctlpre=CI mean=Mean ;
            run;

      /* Calculate HR and 95% CI */
            data &var.;
                        retain variable;
                        set &var.;
                        HR = exp(Mean);
                        HR_CI2_5 = exp(CI2_5);
                        HR_CI97_5 = exp(CI97_5);
                        Variable="&var.";
            run;
%end;

            data cox _final;
                        set &var.;
            run;

%mend cox;

%cox(var = trt);
```
IPCW: inverse probability of censoring weighting.

Supplementary Table S1. Ventilation parameters in the ECMO and conventional MV groups during the first three days of study inclusion.

Variable	All	ECMO	Conventional MV	*P*-value
No. of patients	222	42	180	
Day 1				
Respiratory rate, breaths/min	24 (21–28)	26 (22–29)	24 (21–27)	0.08
Tidal volume, mL/kg PBW	7.3 (6.3–8.4)	6.8 (5.9–7.6)	7.4 (6.4–8.5)	0.006
Minute ventilation, L/min*	10.4 (8.7–12.3)	11.2 (9.5–12.7)	10.3 (8.7–12.1)	0.13
PEEP, cmH_2O	10 (8–12)	10 (8–12)	10 (8–11)	0.55
PIP, cmH_2O	26 (24–30)	26 (23–29)	27 (24–30)	0.25
Missing data	5 (2.3)	1 (2.4)	4 (2.2)	
Driving pressure, cmH_2O†	16 (14–20)	15 (14–18)	16 (14–20)	0.14
Missing data	5 (2.3)	1 (2.4)	4 (2.2)	
Compliance, mL/cmH_2O‡	27.1 (21.0–32.5)	28.5 (22.5–33.8)	26.1 (20.1–32.5)	0.23
Missing data	5 (2.3)	1 (2.4)	4 (2.2)	
Mechanical power, J/min§	32.2 (26.2–39.1)	30.8 (24.6–37.2)	32.2 (26.3–39.2)	0.21
Missing data	5 (2.3)	1 (2.4)	4 (2.2)	
Day 2				
Respiratory rate, breaths/min	24 (20–27)	16 (13–20)	25 (22–28)	< 0.001
Missing data	15 (6.8)	3 (7.1)	12 (6.7)	
Tidal volume, mL/kg PBW	7.0 (6.1–8.2)	5.1 (3.9–6.5)	7.3 (6.4–8.7)	< 0.001
Missing data	17 (7.7)	5 (11.9)	12 (6.7)	
Minute ventilation, L/min*	10.2 (8.1–11.8)	5.1 (3.7–7.7)	10.7 (9.1–12.1)	< 0.001
Missing data	17 (7.7)	5 (11.9)	12 (6.7)	
PEEP, cmH_2O	10 (8–11)	8 (7–12)	10 (8–11)	0.30
Missing data	15 (6.8)	3 (7.1)	12 (6.7)	
PIP, cmH_2O	25 (22–28)	21 (19–24)	26 (23–30)	< 0.001
Missing data	21 (9.5)	5 (11.9)	16 (8.9)	
Driving pressure, cmH_2O†	15 (12–18)	11 (10–13)	16 (13–19)	< 0.001
Missing data	21 (9.5)	5 (11.9)	16 (8.9)	
Compliance, mL/cmH_2O‡	27.4 (20.8–35.0)	28.2 (21.6–35.9)	27.3 (20.6–35.0)	0.53
Missing data	23 (10.4)	7 (16.7)	16 (8.9)	
Mechanical power, J/min§	29.8 (22.0–36.2)	11.6 (8.4–15.9)	32.3 (27.0–38.3)	< 0.001
Missing data	23 (10.4)	7 (16.7)	16 (8.9)	
Day 3				
Respiratory rate, breaths/min	23 (20–27)	16 (13–20)	24 (22–28)	< 0.001
Missing data	26 (11.7)	3 (7.1)	23 (12.8)	
Tidal volume, mL/kg PBW	7.0 (6.1–8.1)	4.6 (3.8–6.4)	7.3 (6.6–8.3)	< 0.001
Missing data	28 (12.6)	5 (11.9)	23 (12.8)	
Minute ventilation, L/min*	10.1 (8.2–11.6)	4.7 (3.7–7.0)	10.4 (9.2–12.1)	< 0.001
Missing data	28 (12.6)	5 (11.9)	23 (12.8)	
PEEP, cmH_2O	10 (8–11)	10 (7–12)	10 (8–10)	0.81
Missing data	26 (11.7)	3 (7.1)	23 (12.8)	
PIP, cmH_2O	25 (22–28)	22 (17–24)	25 (22–28)	< 0.001
Missing data	32 (14.4)	5 (11.9)	27 (15.0)	
Driving pressure, cmH_2O†	15 (12–18)	11 (9–14)	15 (13–20)	< 0.001
Missing data	32 (14.4)	5 (11.9)	27 (15.0)	
Compliance, mL/cmH_2O‡	31.2 (24.7–39.0)	25.8 (21.3–34.5)	27.7 (21.2–33.9)	0.79
Missing data	34 (15.3)	7 (16.7)	27 (15.0)	
Mechanical power, J/min§	28.5 (21.4–35.6)	11.5 (6.8–16.4)	30.5 (26.1–36.9)	< 0.001
Missing data	34 (15.3)	7 (16.7)	27 (15.0)	

Data are presented as medians (interquartile ranges) or percentages (including a category for missing data). *Calculated as respiratory rate × tidal volume. †Calculated as PIP – PEEP. ‡Calculated as tidal volume/driving pressure. §Calculated as 0.098 × tidal volume × respiratory rate × (PIP – 1/2 × driving

pressure). ECMO: extracorporeal membrane oxygenation; MV: mechanical ventilation; PBW: predicted body weight; PEEP: positive end-expiratory pressure; PIP: peak inspiratory pressure.

Supplementary Table S2. Chest CT scans and pulmonary function tests in the ECMO and conventional MV groups at follow-up.

Variable	All	ECMO	Conventional MV	*P*-value
**Chest CT scans**				
No. of patients	79	14	65	
Time from ICU admission to CT scan, days	48 (28–74)	62 (33–87)	47 (25–64)	0.07
Reticulation	20 (25.3)	5 (35.7)	15 (23.1)	0.33
Architectural distortion	36 (45.6)	4 (28.6)	32 (49.2)	0.16
Traction bronchiectasis	50 (63.3)	10 (71.4)	40 (61.5)	0.37
Honeycombing	4 (5.1)	1 (7.1)	3 (4.6)	0.55
**Pulmonary function tests***				
No. of patients	27	8	19	
FEV_1	79.0 (62.0–86.0)	68.0 (55.5–84.5)	81.0 (68.8–86.5)	0.18
FVC	67.0 (54.0–79.0)	60.5 (41.5–79.0)	74.0 (55.5–80.0)	0.33
FEV_1/FVC	86.0 (82.5–89.5)	87.5 (84.5–90.5)	85.8 (82.0–88.0)	0.49
DLCO	52.0 (42.0–68.0) [n = 22]	47.5 (44.0–59.0) [n = 6]	55.5 (41.5–68.5) [n = 16]	0.42
Vital capacity	80.0 (66.5–94.0) [n = 16]	58.0 (50.5–66.5) [n = 3]	83.0 (79.0–95.0) [n = 13]	0.04

Data are presented as medians (interquartile ranges) or percentages. *% of predicted. CT: computed tomography; DLCO: diffusing capacity; ECMO: extracorporeal membrane oxygenation; ICU: intensive care unit; MV: mechanical ventilation.

Supplementary Table S3. Baseline characteristics of IPCW-weighted cohort.

Variable	All	ECMO	Conventional MV	SMD
No. of patients	543	42	501	
Age, years	73 (64–76)	61 (49–68)	73 (66–76)	–0.95
Sex				0.35
Male	379 (69.8)	35 (83.3)	344 (68.7)	
Female	164 (30.2)	7 (16.7)	157 (31.3)	
Body mass index, kg/m^2	24.2 (22.3–26.6)	25.6 (22.8–27.4)	24.1 (22.3–26.6)	0.37
Charlson Comorbidity Index	1 (0–2)	1 (0–2)	1 (0–2)	0.04
Type of oxygen support				0.24
Mask with reservoir bag	394 (72.6)	28 (66.7)	366 (73.1)	
High-flow nasal cannula	68 (12.5)	8 (19.0)	60 (12.0)	
Intubated state	42 (7.7)	4 (9.5)	38 (7.6)	
SOFA score	4 (3–6)	4 (3–9)	4 (3–6)	0.38
Laboratory findings				
Lymphocyte, %	6.5 (4.0–12.0)	7.2 (4.1–10.5)	6.2 (4.0–12.5)	–0.08
Platelet count, $1000/mm^3$	178 (146–259)	208 (146–288)	172 (146–251)	0.25
Total bilirubin, mg/dL	0.5 (0.4–0.8)	0.6 (0.5–0.8)	0.5 (0.4–0.8)	0.12
Creatinine, mg/dL	0.9 (0.6–1.1)	0.9 (0.6–1.2)	0.9 (0.6–1.1)	0.15
PaO_2/FiO_2*	65 (58–76)	78 (57–108)	65 (59–76)	0.50
$PaCO_2$, mmHg*	47 (39–61)	47 (37–55)	47 (39–62)	–0.23
Bicarbonate, mEq/L	22.6 (20.5–25.1)	22.5 (20.8–24.0)	22.6 (20.4–25.1)	–0.06
Time from ICU admission to MV initiation, days	1 (0–7)	0 (0–1)	1 (0–4)	–0.51
Corticosteroids	533 (98.2)	40 (95.2)	493 (98.4)	–0.18
Neuromuscular blocker	501 (92.3)	36 (85.7)	465 (92.8)	–0.23
Inhaled nitric oxide	249 (45.9)	10 (23.8)	239 (47.7)	–0.51
Prone positioning	384 (70.7)	18 (42.9)	366 (73.1)	–0.64
Renal replacement therapy	127 (23.4)	19 (45.2)	108 (21.6)	0.52
MV parameters*				
Respiratory rate, breaths/min	26 (23–30)	26 (22–29)	26 (23–30)	–0.18
Tidal volume, mL/kg PBW	7.7 (6.6–9.6)	6.8 (5.9–7.6)	7.8 (6.6–9.9)	–0.70
Minute ventilation, L/min†	11.9 (9.9–14.9)	11.1 (9.5–12.7)	12.0 (9.9–15.0)	–0.44
PEEP, cmH_2O	9 (7–10)	10 (8–12)	9 (7–10)	0.39
PIP, cmH_2O	26 (23–30)	26 (23–29)	26 (23–30)	–0.13
Driving pressure, cmH_2O‡	18 (14–20)	16 (14–18)	18 (14–21)	–0.22
Compliance, mL/cmH_2O§	27.7 (20.9–35.6)	28.0 (22.5–33.8)	27.6 (20.5–35.6)	0.04
Mechanical power, J/min¶	37.4 (28.6–45.9)	30.8 (24.6–37.2)	38.5 (29.1–46.7)	–0.47

Data are presented as medians (interquartile ranges) or percentages. *Assessed on the day of study inclusion. †Calculated as respiratory rate × tidal volume. ‡Calculated as PIP – PEEP. §Calculated as tidal volume/driving pressure. ¶Calculated as 0.098 × tidal volume × respiratory rate × (PIP – 1/2 × driving pressure). ECMO: extracorporeal membrane oxygenation; FiO_2: fraction of inspired oxygen; IPCW: inverse probability of censoring weighting; MV: mechanical ventilation; $PaCO_2$: arterial carbon dioxide tension; PaO_2: arterial oxygen tension; PBW: predicted body weight; PEEP: positive end-expiratory pressure; PIP: peak inspiratory pressure; SMD: standardized mean difference; SOFA: Sequential Organ Failure Assessment.

Supplementary Table S4. Incidence rates and hazard ratios comparing hospital mortality between ECMO and conventional MV according to subgroup.

Group	Incidence rate per 100 person-days (95% CI)	Hazard ratio (95% CI)
**Age ≥ 70 years**		
Before weighting		
ECMO	1.53 (0.87–2.29)	0.56 (0.35–0.86)
Conventional MV	2.40 (2.14–2.66)	
IPCW-weighted		
ECMO	1.86 (0.94–2.76)	0.61 (0.26–4.06)*
Conventional MV	3.56 (3.06–4.13)	
**Age < 70 years**		
Before weighting		
ECMO	0.95 (0.64–1.38)	0.78 (0.51–1.21)
Conventional MV	1.17 (0.98–1.38)	
IPCW-weighted		
ECMO	0.99 (0.54–1.52)	0.28 (0.09–0.71)*
Conventional MV	1.84 (1.40–3.17)	
**Charlson Comorbidity Index ≥ 2**		
Before weighting		
ECMO	1.71 (0.81–3.17)	0.76 (0.38–1.48)
Conventional MV	2.19 (1.83–2.62)	
IPCW-weighted		
ECMO	1.88 (0.86–3.35)	1.15 (0.36–4.65)*
Conventional MV	2.89 (2.20–4.16)	
**Charlson Comorbidity Index < 2**		
Before weighting		
ECMO	0.92 (0.60–1.29)	0.48 (0.32–0.72)
Conventional MV	1.81 (1.64–1.98)	
IPCW-weighted		
ECMO	0.96 (0.54–1.43)	0.50 (0.28–0.90)*
Conventional MV	2.88 (2.50–3.32)	
**Prone positioning**		
Before weighting		
ECMO	0.89 (0.36–1.47)	0.37 (0.16–0.64)
Conventional MV	2.17 (1.95–2.41)	
IPCW-weighted		
ECMO	0.96 (0.36–1.65)	0.28 (0.11–0.75)*
Conventional MV	3.34 (2.89–3.89)	
**No prone positioning**		
Before weighting		
ECMO	1.24 (0.80–1.84)	0.83 (0.52–1.31)
Conventional MV	1.46 (1.22–1.80)	
IPCW-weighted		
ECMO	1.31 (0.84–1.95)	1.18 (0.58–2.94)*
Conventional MV	2.11 (1.61–3.45)	
**PaO_2/FiO_2 ≥ 65**		
Before weighting		
ECMO	0.68 (0.41–1.03)	0.41 (0.26–0.64)
Conventional MV	1.55 (1.37–1.76)	
IPCW-weighted		
ECMO	0.72 (0.40–1.13)	0.52 (0.26–1.52)*
Conventional MV	2.17 (1.81–2.60)	
**PaO_2/FiO_2 < 65**		
Before weighting		

ECMO	2.16 (1.48–3.36)	0.80 (0.52–1.39)
Conventional MV	2.49 (2.20–2.81)	
IPCW-weighted		
ECMO	2.44 (1.66–3.69)	0.49 (0.25–1.31)*
Conventional MV	4.18 (3.52–5.64)	
**Driving pressure ≥ 15 cmH_2O**		
Before weighting		
ECMO	0.94 (0.62–1.37)	0.41 (0.27–0.58)
Conventional MV	2.14 (1.94–2.36)	
IPCW-weighted		
ECMO	1.01 (0.57–1.60)	0.28 (0.17–0.44)*
Conventional MV	3.42 (2.92–4.19)	
**Driving pressure < 15 cmH_2O**		
Before weighting		
ECMO	1.48 (0.81–2.87)	1.10 (0.61–2.35)
Conventional MV	1.37 (1.13–1.72)	
IPCW-weighted		
ECMO	1.56 (0.78–3.02)	5.10 (1.78–28.20)*
Conventional MV	1.76 (1.43–2.20)	

*Adjusted for age, sex, body mass index, type of oxygen support, SOFA score, PaO_2/FiO_2, $PaCO_2$, time from ICU admission to MV initiation, neuromuscular blocker, inhaled nitric oxide, prone positioning, renal replacement therapy, and mechanical power. CI: confidence interval; ECMO: extracorporeal membrane oxygenation; FiO_2: fraction of inspired oxygen; IPCW: inverse probability of censoring weighting; MV: mechanical ventilation; PaO_2: arterial oxygen tension.

Supplementary Table S5. Incidence rates and hazard ratios comparing fibrotic changes between ECMO and conventional MV, in the model including only patients who survived to hospital discharge.

Group	Incidence rate per 100 person-days (95% CI)	Hazard ratio (95% CI)
Before weighting		
ECMO	0.68 (0.38–1.10)	0.47 (0.26–0.96)
Conventional MV	1.04 (0.87–1.20)	
IPCW-weighted		
ECMO	0.67 (0.33–1.11)	0.29 (0.05–1.14)*
Conventional MV	1.15 (0.94–1.39)	

*Adjusted for age, sex, body mass index, type of oxygen support, SOFA score, PaO_2/FiO_2, $PaCO_2$, time from ICU admission to MV initiation, neuromuscular blocker, inhaled nitric oxide, prone positioning, renal replacement therapy, and mechanical power. CI: confidence interval; ECMO: extracorporeal membrane oxygenation; IPCW: inverse probability of censoring weighting; MV: mechanical ventilation.

Supplementary Table S6. Incidence rates and hazard ratios comparing hospital mortality between ECMO and conventional MV, in the model including patients who received ECMO and lung transplant.

Group	Incidence rate per 100 person-days (95% CI)	Hazard ratio (95% CI)
Before weighting		
ECMO	0.96 (0.67–1.32)	0.52 (0.36–0.73)
Conventional MV	1.87 (1.72–2.05)	
IPCW-weighted		
ECMO	1.01 (0.65–1.44)	0.51 (0.33–0.89)*
Conventional MV	2.84 (2.47–3.39)	

*Adjusted for age, sex, body mass index, type of oxygen support, SOFA score, PaO_2/FiO_2, $PaCO_2$, time from ICU admission to MV initiation, neuromuscular blocker, inhaled nitric oxide, prone positioning, renal replacement therapy, and mechanical power. CI: confidence interval; ECMO: extracorporeal membrane oxygenation; IPCW: inverse probability of censoring weighting; MV: mechanical ventilation.

Supplementary Table S7. Incidence rates and hazard ratios comparing hospital mortality or fibrotic changes between ECMO and conventional MV, in the model further adjusted for the wild type and Alpha/Delta periods.

Group	Incidence rate per 100 person-days (95% CI)	Hazard ratio (95% CI)
**Hospital mortality**		
Before weighting		
ECMO	1.07 (0.74–1.42)	0.54 (0.37–0.75)
Conventional MV	1.92 (1.76–2.09)	
IPCW-weighted		
ECMO	1.13 (0.75–1.58)	0.55 (0.35–0.97)*
Conventional MV	2.88 (2.51–3.43)	
**Fibrotic changes**		
Before weighting		
ECMO	0.61 (0.33–0.94)	0.44 (0.24–0.84)
Conventional MV	0.97 (0.82–1.12)	
IPCW-weighted		
ECMO	0.60 (0.31–0.94)	0.18 (0.03–0.55)*
Conventional MV	1.15 (0.96–1.34)	

*Adjusted for age, sex, body mass index, type of oxygen support, SOFA score, PaO_2/FiO_2, $PaCO_2$, time from ICU admission to MV initiation, neuromuscular blocker, inhaled nitric oxide, prone positioning, renal replacement therapy, mechanical power, and study period (Feb 2020–Jun 2021 vs Jul–Dec 2021).
CI: confidence interval; ECMO: extracorporeal membrane oxygenation; IPCW: inverse probability of censoring weighting; MV: mechanical ventilation.

Supplementary Table S8. Hospital mortality and fibrotic changes between the ECMO and conventional MV groups.

Group	%
**Hospital mortality**	
Before weighting	
ECMO	53.7
Conventional MV	71.3
IPCW-weighted	
ECMO	51.5
Conventional MV	70.6
**Fibrotic changes**	
Before weighting	
ECMO	42.9
Conventional MV	44.8
IPCW-weighted	
ECMO	43.0
Conventional MV	45.0

ECMO: extracorporeal membrane oxygenation; IPCW: inverse probability of censoring weighting; MV: mechanical ventilation.

Supplementary Figure S1. Survival from hospital admission to day 60 by study group in the IPCW-weighted cohort

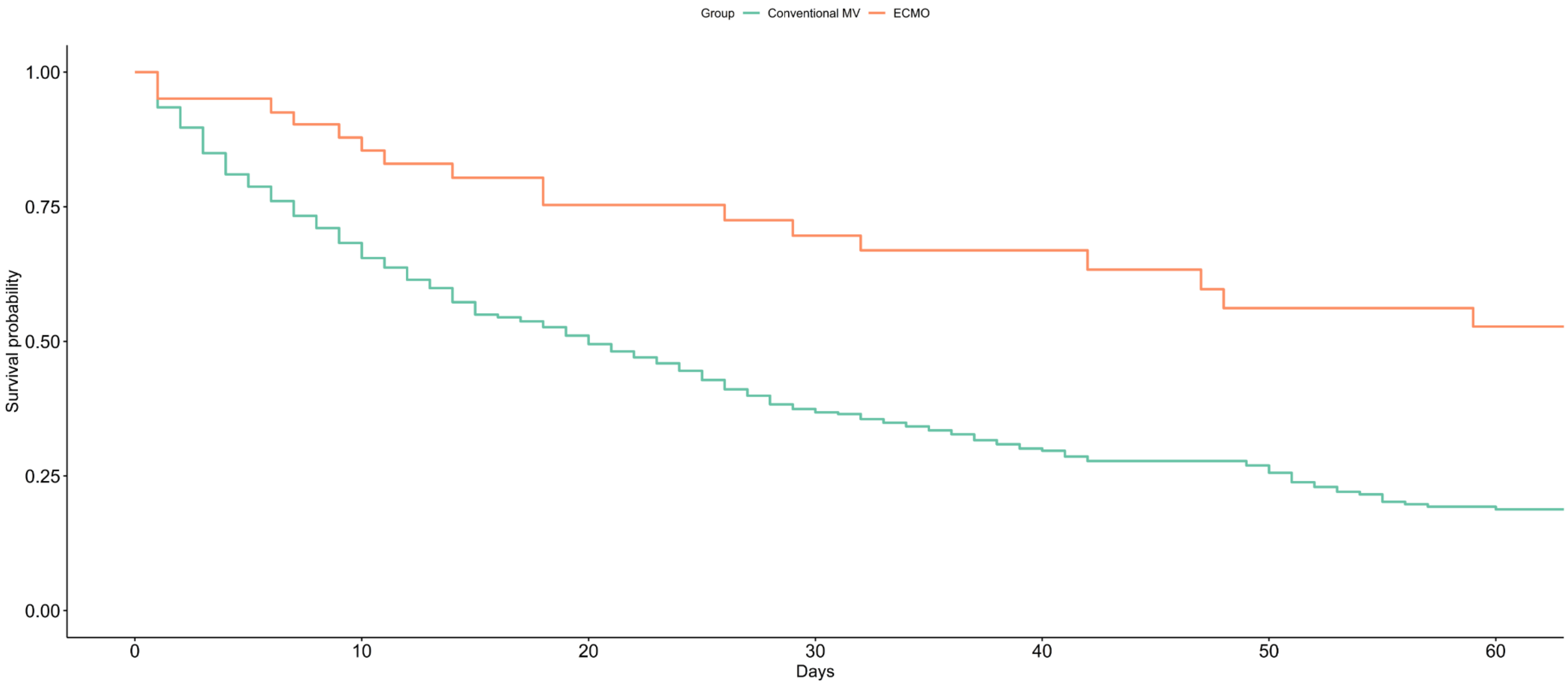

ECMO: extracorporeal membrane oxygenation; IPCW: inverse probability of censoring weighting; MV: mechanical ventilation.
